# Sex steroid priming for growth hormone stimulation testing in children and adolescents with short stature: A systematic review

**DOI:** 10.1111/cen.14862

**Published:** 2022-12-22

**Authors:** Gregor Duncan, Sarah Kiff, Rod T. Mitchell

**Affiliations:** ^1^ Royal Hospital for Children and Young People Edinburgh UK; ^2^ MRC Centre for Reproductive Health, Queen's Medical Research Institute The University of Edinburgh Edinburgh UK

**Keywords:** children, growth hormone, insulin tolerance test, oestrogen, peripuberty, sex steroid, testosterone

## Abstract

**Objective:**

Growth hormone stimulation testing (GHST) is used to diagnose growth hormone deficiency (GHD) in children. As sex steroids impact on anterior pituitary function, there is concern around the efficacy of GHST in peripubertal children, where endogenous sex steroid levels are low. Sex steroid priming before GHST is thought to improve test efficacy in these children, however evidence to support its use in clinical practice is limited.

In this systematic review, we addressed the following research questions: Does priming increase GH stimulation test efficacy in peripubertal children? Does priming identify those who would benefit most from treatment in terms of final height? Is there evidence for an optimal sex‐steroid priming regimen?

**Design, Patients, Measurements:**

The study was registered with PROSPERO and conducted according to PRISMA guidelines. We searched Medline, Cochrane‐Library, Scopus, EMBASE and Web‐of‐Science and included all studies that included GHST in both primed and unprimed children. A GH cut‐off of 7 µg/L was used as a threshold for GHD. Study quality was assessed using the Risk‐Of‐Bias in Non‐ Randomized Studies (ROBINS‐I) tool or the revised Cochrane risk‐of‐bias tool for Randomised trials.

**Results:**

Fifteen studies met our inclusion criteria, of which 4/15 (27%) were randomised control trials. The majority (9/15) of the studies indicated that priming increases growth hormone response upon GHST in peripubertal children, increasing test specificity. Two studies investigated final height after treatment based on the results of primed versus unprimed GHST. These results indicate that growth hormone treatment based on results of a primed GHST improve outcomes compared with treatment based on an unprimed test.

**Conclusion:**

Sex‐steroid priming increases the growth hormone response during GHST, resulting in fewer patients meeting the threshold required for a diagnosis of GHD. Unnecessary GH treatment may be avoided in some patients without a detrimental effect on final height. Numerous sex‐steroid priming regimens have been used in clinical practice and the majority appear to be effective, but an optimal regimen has not been determined.

## 1 INTRODUCTION

Children presenting in the peripubertal period with short stature, decreased growth velocity or failure to undergo a pubertal growth spurt require endocrine evaluation. In such cases differentiating growth‐hormone deficiency (GHD) from non‐GH‐deficient constitutional delay of growth and puberty (CDGP), idiopathic short stature (ISS) and other causes of short stature is required. Growth hormone stimulation tests (GHST) are required to diagnose GHD and are based on the concept of a pharmacological agent acutely stimulating pituitary GH secretion. Accurate diagnosis of GHD and treatment with recombinant human growth hormone (r‐GH) is important to achieve adult height (AH) potential.^1^ Conversely re‐testing of children identified as GHD when they reach final adult height, often reveals GH sufficiency,^2^, ^3^, ^4^ questioning the accuracy of the original diagnosis and the impact of GH treatment.

Sex steroids may modulate GH secretion and have the potential to impact the results of GHST.^5^ In males, testosterone has been shown to stimulate growth hormone secretion, at least in part due to its aromatization to oestradiol.^6^ In females, the effect of oestrogen on growth hormone production is less clear. It may have a direct effect on the pituitary stimulating GH production (although mechanisms of this remains uncertain) and indirect effects on GH production by reducing hepatic IGF‐1 production resulting in secondary increase in GH secretion.^7^, ^8^

It is therefore important to consider that in the peripubertal age‐range, prepubertal patients with low levels of circulating sex steroids may have a blunted response to GHST than pubertal age‐matched peers. In these children, priming, which involves administering exogenous sex steroids before testing to sensitise the pituitary gland and potentiate GH secretion during GHST, can be performed. Even in children and adolescents with normal stature or idiopathic short stature, GH responses to various stimuli can be inconsistent and frequently do not reach the conventional cut‐offs of 7 or 10 μg/L.^9^ In addition, unprimed GHST have been reported to have poor specificity for diagnosing GHD in this cohort, while primed tests have been shown to reduce false‐positive diagnoses of GHD.^10^, ^11^, ^12^, ^13^

The use of priming is still a matter of debate. The potential benefits that priming increases GHST specificity, reducing the rate of false positive results must be weighed against the concept that priming can affect physiology, producing only a transient rise in GH secretion, and that children who might benefit from GH therapy could be missed.^14^, ^15^

Guidelines on the diagnosis and management of GHD in children and adolescents have placed emphasis on avoiding unnecessary GH treatment in healthy patients with ISS and/or CDGP, considering its potential harms, costs and physical/psychological burden.^16^, ^17^ Guidelines specifically recommending priming in short (adult height [AH] prognosis<‐2 SDS) prepubertal boys aged over 11 and girls aged over 10, aiming to prevent unnecessary r‐GH treatment in those where CDGP is likely have been produced,^16^ whilst others were not able to reach consensus on the use of sex steroid priming in GHST.^17^ It has also been suggested that priming may improve GHST specificity in prepubertal children of any age.^11^, ^12^, ^18^ In addition to the issue of specificity, a recent audit has shown that in clinical practice, the regimens used for priming vary significantly.^19^

Despite the importance of accurate diagnosis and treatment of GHD a systematic review of the evidence for sex‐steroid priming for GHST has not previously been conducted. Therefore, the aim of our study was to perform a systematic review and meta‐analysis of the literature, focusing on the impact of sex‐steroid priming on the results of GHST and the influence of treatment based on a ‘primed’ or ‘unprimed’ test on final height.

## 2 METHODS

We performed a comprehensive search of published clinical studies involving sex‐steroid priming for GHST. This review is reported according to the PRISMA guidelines for systematic reviews.^20^ The study protocol was registered as a systematic review on Prospero (registration number CRD42021244443) and is available from: https://www.crd.york.ac.uk/prospero/display_record.php?ID=CRD42021244443.

### 2.1 Information source

The search was included the following databases: Medline, Cochrane Library, Scopus, EMBASE and Web of Science.

### 2.2 Inclusion criteria

Inclusion criteria for study selection were:

- Articles published between January 1, 1900 and November 20, 2020 and written in English;
- Participants were Children (0–9 years) and/or adolescents (10–19 years);
- Includes ‘primed’ and ‘unprimed’ patients
- Outcomes include effect of sex‐steroid priming on outcome of GHST or final adult height

### 2.3 Exclusion criteria

Exclusion criteria for the study selection were:

- Studies not in the English language
- Conference abstracts and reviews

### 2.4 Search and study selection

**Figure 1:**
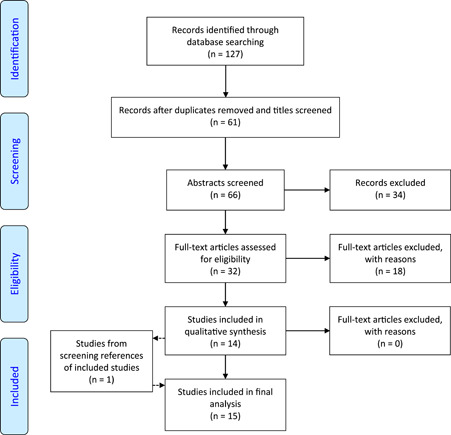
PRISMA flow diagram for study selection^20^ [Color figure can be viewed at wileyonlinelibrary.com]

The search strategy and terms (Supporting Information: Table 1) were adapted from an earlier study. The number of records identified after removal of duplicates was 66; initial screening of titles and abstracts was performed independently by two assessors (GD and RTM) using selection based on inclusion/exclusion criteria. For abstracts that were not selected by both assessors, a final decision on inclusion was made following discussion between assessors. Full texts were obtained for 32 studies and a further 18 articles were excluded as a result of not meeting all of the inclusion criteria (Supporting Information: Table 2). An additional article was identified on screening the reference lists of included papers. Fifteen papers were included in the final analysis. A summary of the selection process is provided in the PRISMA flowchart (Figure 1).

### 2.5 Data extraction and summary measures

The following data—(1) author (2) year of publication (3) study design (4) cohort size (5) sex steroid used (6) GHST used—were extracted separately by GD and RTM. Further data were extracted for data analysis: (7) population characteristics (8) pretest criteria (inclusion criteria for subjects) (9) control group included (10) study results.

### 2.6 Quality assessment, bias assessment and data synthesis

For the methodological evaluation the following aspects were assessed: validity of chosen indicators of pubertal status (Tanner stage, bone age [BA], chronological age [CA], testicular volume), assessment of whether method of GHST was performed according to accepted standard practice, and appropriateness of statistical analysis. Additionally, each study underwent a risk of bias assessment. Risk of bias was assessed using the revised Cochrane risk‐of‐bias tool for Randomised trials (Rob 2) (Supporting Information: Table 3) or the risk of bias in nonrandomized studies—of interventions (ROBINS‐1) tool (Supporting Information: Table 4) according to published guidelines^21^

### 2.7 Statistics

Data for individual studies was represented according to reported mean ± 95% confidence interval and statistical significance was *p* < .05. Data for composite was presented as mean ± SEM and statistical analysis was performed using a Wilcoxon matched‐pairs signed rank test, with statistical significance set at <0.05.

## 3 RESULTS

A summary of the studies included in this systematic review including priming regimen, GHST used, cohort size, population characteristics and results can be seen in Table 1. There was significant heterogeneity in the included study populations, with children in the pre‐ and peri‐pubertal age range (range 1–17 years), Tanner stage 1–5, variation in GHST used (ITT, arginine, glucagon, clonidine, levodopa, ornithine, exercise) and priming regimen used (oral oestrogen, transdermal oestrogen, intramuscular testosterone, mixed regimens). A detailed description of all of the studies can be found in Supporting Information: Table 5, and significant differences between the study populations described are highlighted in the results and discussion.

**Table 1:** Summary of studies included in the review

Study	Study design	Level of evidence^a^	Cohort size	Sex steroid	GHST
Moll et al.^13^	Control study	2	23	Oestrogen	Levodopa
Wilson et al.^22^	RCT	1	65	Oestrogen	Variable: arginine, ITT, clonidine
Chatterjee et al.^23^	Control study	2	28	Testosterone	ITT
Marin et al.^11^	RCT	1	84	Oestrogen	Variable: arginine, ITT, exercise
Martinez et al.^12^	RCT	1	59	Oestrogen	Variable: arginine, clonidine
Gonc et al.^24^	Control study	2	84	Testosterone	Levodopa
Chemaitilly et al.^25^	Retrospective cohort study	3	47	Testosterone	Variable: arginine, ITT, glucagon, ornithine
Muller et al.^26^	Control study	2	26	Testosterone	Arginine
Couto‐Silva et al.^25^	Retrospective cohort study	3	148	Testosterone	Arginine, ITT
Borghi et al.^27^	Control study	2	22	Oestrogen	Clonidine
Gonc et al.^24^	Retrospective cohort study	3	50	Testosterone	Levodopa
Molina et al.^18^	Control study	2	39	Oestrogen (females) testosterone (males)	Clonidine
Soliman et al.^28^	RCT	1	92	Oestrogen (females) testosterone (males)	Clonidine
Sato et al.^29^	Retrospective cohort study	3	3	Testosterone	Variable: arginine, glucagon, ITT
Galazzi et al.^30^	Retrospective cohort study	3	184	Variable–oestrogen, testosterone	Variable: arginine, clonidine, glucagon, ITT

Abbreviations: ITT, insulin tolerance test; RCT, randomised controlled trial.

^a^ Level of evidence (adapted from https://www.cebm.net/2016/05/ocebm-levels-of-evidence/). 1—Properly powered and conducted randomised clinical trial; systematic review with meta‐analysis. 2—Well‐designed controlled study without randomisation; prospective comparative cohort study. 3—Case–control studies; retrospective cohort study. 4—Case series with or without intervention; cross‐sectional study. 5—Opinion of respected authorities; case reports.

Unless otherwise stated a peak GH of 7 μg/L or higher was used as a normal response to GHST in all studies.

### 3.1 Comparison of GH response with and without sex steroid priming: Randomised control trial (RCTs)

**Figure 2:**
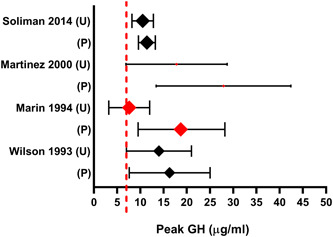
Forest plot for RCTs investigating the impact of sex steroid priming on the results of GHST in children. Peak growth hormone displayed as mean±95% CI. The relative size of each data point indicates the number of patients included in the study. Data points in red indicate a statistically significant difference (*p* < .05) between the primed (P) and unprimed (U) results. Broken red line represents threshold for diagnosis of GHD. ghd, growth hormone deficiency; GHST, Growth hormone stimulation testing; RCTs, randomised control trials. [Color figure can be viewed at wileyonlinelibrary.com]

Four RCTs of GH response to stimulation with and without priming were identified. Results of these are summarised in Figure 2.

Martinez and colleagues randomised 15 prepubertal children with GHD and 44 short non‐GHD children to oestrogen priming or placebo before sequential arginine‐clonidine test. There was an increased GH response in primed non‐GHD children versus placebo, but no significant effect of priming on GH response in the GHD group. Priming improved the discrimination power of GHST between short non‐GHD and GHD children.^12^ Mean peak GH response following GHST was 17.8 ± 10.9 µg/L in the placebo group and 27.9 ± 14.5 µg/L in the oestrogen primed group in non‐GHD children (*p* = .0001).

Marin and colleagues assessed 84 normal height children at different pubertal stages in whom there was no clinical suspicion of GHD. Treadmill exercise, arginine and insulin test were performed, with children randomised to priming or no priming. They found that priming with oestrogen increased mean peak GH response from 7.6 ± 4.4 µg/L to 18.7 ± 9.2 µg/L (*p* < .05).^11^ Considering unprimed patients, 44% children at Tanner stage 2 and 11% at Tanner stage 3 would be considered GH deficient, whilst with priming, all children reached a peak GH response >7 µg/L.

Wilson et al carried out a RCT of 65 pre/peri‐pubertal children with growth failure and reported that priming with conjugated oestrogen did not enhance the efficacy of clonidine GHST,^22^ albeit with a defined normal GH response at 10 mcg/L, higher than that utilised in most studies. There were no statistically significant differences in the mean peak GH response (16.3 ± 8.7 µg/L primed vs. 14.0 ± 7.5 µg/L control; *p* > .05).

Soliman and colleagues performed a RCT assessing 92 prepubertal children with idiopathic short stature, randomised to priming (conjugated oestrogen or testosterone) or no priming. Priming before clonidine GHST did not increase the mean peak GH response (11.4 ± 1.8 µg/L primed vs. 10.5 ± 2.3 µg/L unprimed; *p* > .05).^28^

### 3.2 Comparison of GH response with and without sex steroid priming: Non‐RCTs

**Figure 3:**
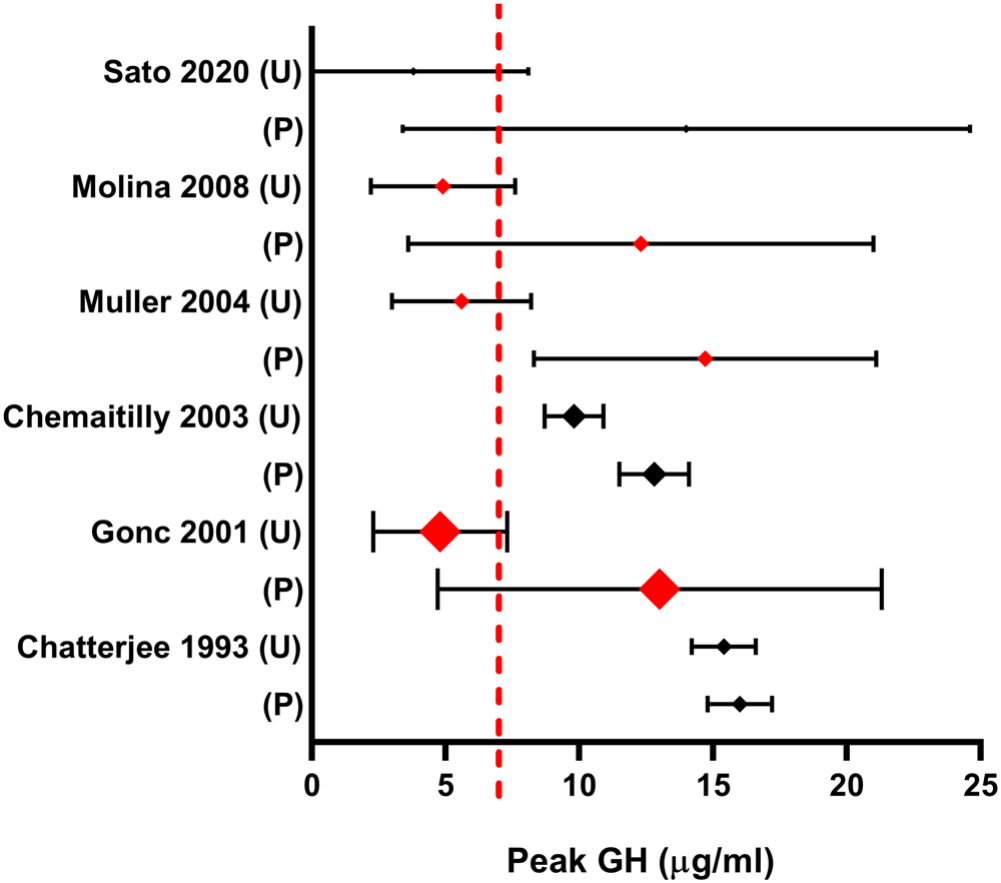
Forest plot for non‐RCTs investigating the impact of sex steroid priming on the results of GHST in children. Peak growth hormone displayed as mean±95% CI. The relative size of each data point indicates the number of patients included in the study. Data points in red indicate a statistically significant difference (*p* < .05) between the primed (P) and unprimed (U) results. Broken red line represents threshold for diagnosis of GHD. GHD, growth hormone deficiency; RCTs, randomised control trials. [Color figure can be viewed at wileyonlinelibrary.com]

Results of non‐randomised studies comparing GH response to stimulation testing in primed and unprimed subjects are summarised in Figure 3. These can be divided into cohort studies of patient that underwent either primed or unprimed GHST, and studies where subjects underwent an unprimed GHST followed by a primed GHST.

Three studies compared GH response between groups of patients that had undergone a single GHST, either primed or unprimed. Moll and colleagues assessed 23 prepubertal short‐normal children with levodopa stimulation test with and without oestrogen priming. They found that 5/23 unprimed tests failed to exceed the normal response cut off (7 µg/L) versus 0/20 primed tests.^13^

A retrospective cohort study carried out by Couto‐Silva and colleagues assessed 148 boys with short stature in whom puberty started after the age of 14 found that mean peak GH response upon GHST was below cut‐off (10 µg/L) in 25% of testosterone primed tests versus 41% of unprimed arginine‐insulin tests (*p* < .05).^31^

A second retrospective cohort study, by Chemaitilly et al.^25^ assessed 47 short prepubertal boys and found that testosterone primed children had higher mean GH during sleep testing (8.1 ± 0.8 µg/L primed vs. 4.7 ± 0.6 µg/L unprimed) (*p* < .05) than unprimed children, but similar mean GH following GHST with arginine‐insulin, glucagon or ornithine stimulation (12.8 ± 1.3 µg/L primed vs. 9.8 ± 1.1 µg/L unprimed; *p* > .05).

Six studies were designed so each subject underwent an unprimed GHST followed by a primed GHST.

Borghi and colleagues carried out unprimed followed by transdermal oestradiol primed clonidine stimulation tests on 34 prepubertal children with familial short stature or CDGP. They found that priming increased mean peak GH response (22 vs. 14.7 µg/L; *p* < .05) upon clonidine GHST.^27^

Gonc and colleagues assessed 84 prepubertal and early‐pubertal short boys who had previously not reached a GH of 10 µg/L on GHST. Priming increased mean peak GH response to levodopa following low dose testosterone (11.5 ± 8.8 µg/L vs. 4.8 ± 2.8 µg/L; *p* < .001) and conventional dose testosterone (13.0 ± 8.3 µg/L vs. 4.8 ± 2.5 µg/L; *p* < .001).^32^

Muller and colleagues repeated arginine GH stimulation testing in 26 boys with short stature and delayed puberty, who had previously had low unprimed GHST responses (defined as peak GH <10 µg/L). Priming with testosterone increased mean peak GH response to arginine stimulation to a level above cut‐off (10 µg/L) in 77% of patients who had previously had an insufficient response (16.8 ± 5.8 µg/L primed vs. 5.6 ± 2.6 µg/L unprimed; *p* < .05).^26^

Molina and colleagues primed 39 children (with testosterone or oestradiol valerate) with delayed puberty and short stature, who had all previously had a low GH response to clonidine (peak GH <10 µg/L). Priming increased mean peak GH response to clonidine stimulation (12.3 ± 8.7 µg/L vs. 4.9 ± 2.7 µg/L; *p* < .05).^18^

Chatterjee et al.^23^ assessed 28 boys with β‐thalassaemia with pubertal failure and found that testosterone priming did not increase mean peak GH response to insulin tolerance test (16.0 ± 1.2 µg/L primed vs. 15.4 ± 0.20 µg/L unprimed; *p* > .05).

Sato et al.^29^ reported a retrospective cohort study of three Japanese prepubertal short boys and showed that testosterone priming increased peak GH response to GHST (Boy 1:4.2 µg/L unprimed arginine vs. 9.2 µg/L primed arginine, Boy 2: 1.9 µg/L unprimed arginine vs. 17.3 µg/L primed arginine, Boy 3:5.3 µg/L unprimed arginine vs. 15.5 µg/L primed glucagon).

### 3.3 Comparison of GH response with and without sex steroid priming: All studies

**Figure 4:**
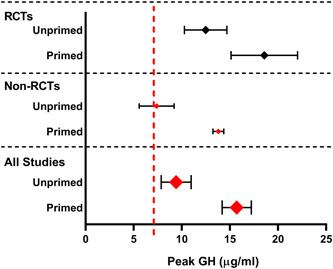
Composite summary of RCTs, Non‐RCTs and All Studies investigating the impact of sex steroid priming on the results of GHST in children. Peak growth hormone displayed as mean ± SEM. The relative size of each data point indicates the number of patients included in each category. Data points in red indicate a statistically significant difference (*p* < .05) between the primed (P) and unprimed (U) results. Broken red line represents threshold for diagnosis of GHD. GHD, growth hormone deficiency; GHST, Growth hormone stimulation testing; RCTs, randomised control trials. [Color figure can be viewed at wileyonlinelibrary.com]

The composite results of RCTs, non‐RCTs and all studies are presented in Figure 4, with data from all studies combined showing higher peak GH response in the primed group (*p* < .05).

### 3.4 Effect of sex steroid dose used for priming on GH response to stimulation

Three studies included an evaluation of the number of doses used during priming on GH response to stimulation testing. Supporting Information: Table 6 provides a summary of the findings, the priming regimen, GHST used, cohort size and population characteristics of each study.

Gonc and colleagues assessed 21 prepubertal or early pubertal boys who had failed to exceed a peak GH above 10 µg/L upon single dose (low or conventional dose) testosterone priming. They found that multiple dose testosterone priming before repeat levodopa GHST increased mean peak GH response compared to single dose priming (10.2 ± 6.1 µg/L vs 5.4 ± 2.5 µg/L; *p* = .004).^32^

Moll and colleagues assessed 23 prepubertal short‐normal children and found that 18/23 achieved a peak GH to levodopa of >7 µg/L on unprimed testing. Priming with 1 or 2 days of oestrogen doses (or variable doses) resulted in 20/20 children reaching a peak GH about threshold of 7 µg/L. The authors comment that there was a similar response to 1 or 2 days of oestrogen treatment, but do not report this data.

Couto‐Silva et al's.^31^ retrospective cohort study assessing boys with short stature with CDGP found that mean peak GH response to arginine‐insulin stimulation test increased in boys given four doses of 100 mg intramuscular testosterone (21.3 ± 2.0 µg/L) compared to those given two doses (14.7 ± 1.7 µg/L; *p* = .04).

### 3.5 Auxological outcomes in primed versus unprimed individuals

The final two studies assessed auxological outcome of children undergoing primed or unprimed GHST.

Galazzi et al.^30^ reported results of a multicentre retrospective cohort study investigating the auxological outcomes of pre/peri‐pubertal children diagnosed with CDGP or GHD who underwent primed vs unprimed GHST and were followed to final height. GHST and priming regimen varied according to local preference. Those with treated GHD diagnosed upon primed GHST reached a greater final height (FH) compared to those with untreated CDGP (*p* = .023). Conversely, in those diagnosed upon an unprimed GHST, no differences in auxological outcomes were identified between treated GHD and untreated CDGP (*p* > .05). These data suggest that priming plays a key role in selecting children who may benefit most from recombinant GH treatment.

Gonc et al.^24^ also reported a retrospective cohort study assessing the auxological outcomes of 50 untreated pre/peri‐pubertal boys with short stature and delayed bone age who had failed to respond to unprimed GHST but responded to primed levodopa GHST, with low dose, conventional dose, or multidosed testosterone. Mean final height of the study group (−1.27 ± 0.72 SDS) was similar to the mid‐parental height (−1.38 ± 0.72 SDS). These data suggest that priming helped identify those in which r‐GH treatment would have been unnecessary as they achieved their adult height potential without GH treatment.

## 4 DISCUSSION

Through their role in sensitising the pituitary axis, priming with sex steroids is considered in those who may display blunted GH response upon unprimed GHST due to the low levels of circulating sex steroids seen in the pre/peri‐pubertal period.

This systematic review identified 15 studies that met our inclusion criteria, of which 4/15 (27%) were RCTs. The majority (9/15) of the studies indicated that priming increases growth hormone response upon GHST in peripubertal children, increasing test specificity. Of these nine studies, three also demonstrated that priming altered the result from an insufficient GH response, to adequate GH response, therefore resulting in fewer subjects meeting the criteria for GH treatment.

Conflicting results, as to the effects of priming on GHST, have also been shown.^23^, ^25^, ^28^ The two RCTs showing no significant difference between peak GH response both utilized clonidine for GH stimulation, raising the question as to whether effect of priming varies depending on GHST used. However, further studies^27^ did find a significant increase in peak GH following primed clonidine tests. Interestingly, the dose of testosterone utilised by Soliman et al. was the lowest seen in all studies included in this review (Supporting Information: Table 5), which may offer an explanation for their results showing no significant effect of testosterone priming.^28^ The three studies addressing dosing regimen for sex steroid priming all concluded that the GH response to GHST was significantly increased with the higher dose sex steroid priming.^13^, ^31^, ^32^ The age of children included in the Soliman et al study was slightly younger than those in other cohorts, and given that advancing Tanner stage alone was shown by Marin and colleagues to increase the percentage of children reaching adequate peak GH response, this is also likely to have influenced differential effect of priming in this study.

Chatterjee et al.^23^ also showed that testosterone priming had no effect on GH response upon GHST, but was the only study to investigate boys with chronic disease (β‐thalassaemia with pubertal failure), limiting the relevance of this data with respect to clinical practice.

Overall, studies that showed an effect of priming tended to be a higher level of evidence with low‐moderate risk of bias. No studies reported a reduction in GH response following priming and no significant adverse effects of priming were reported in these studies. Therefore, it can be concluded the priming before GH stimulation testing had the potential to increase peak GH result sufficiently to alter test outcome and subsequent clinical management.

It is important to consider the impact that priming before GHST has on auxological outcomes. For example, does priming allow patients who would benefit from GH treatment to be identified, and those that would not benefit (e.g., CDGP) to be appropriately not treated. The two studies investigating final height after treatment based on the results of primed versus unprimed GHST both indicate that growth hormone treatment based on results of a primed GHST improve outcomes compared with treatment based on an unprimed test.^24^, ^30^

The major limitation in comparing the studies assessed in this review are that the study designs vary significantly. Population characteristics vary in terms of age, height and pubertal status (chronological age [CA], Tanner stage, BA, testicular volumes, auxological data). Pre‐ and peri‐pubertal definitions are made clinically, biochemically or radiologically in varying combinations across the studies, and therefore it is hard to be certain that the study populations are equivalent. The study protocols included in this systematic review including a wide range of GHSTs (insulin, clonidine, arginine, levodopa, ornithine, exercise, glucagon), sex steroid used for priming and dosing regimens. Additionally, variability in how GH response data were reported limited comparison. More fundamentally, defining a normal GH response threshold is also unclear. For the purpose of this review, a threshold of 7 ug/L has been used by convention of the authors and as the most commonly reported amongst the studies, however a threshold of 10 ug/L is also frequently used. This heterogeneity in study design meant analysis for specific stimulation tests, priming protocols and age range of subjects was not possible.

It is unclear at which pubertal stages young people would benefit from priming that is, would there be benefit in priming all children within the expected pubertal age range. Marin et al.^11^ attempted to identify whether GH response varies with stage of puberty and therefore threshold for normal response to GHST should vary throughout puberty. Priming with oestrogen in the pre‐pubertal groups increased the GH response in all children above the 7 µg/L GH peak threshold. There was a significant increase in the percentage of healthy children reaching the threshold of 7 µg/L peak GH to unprimed GHST with advancing pubertal stage, indicating that priming might be unnecessary when Tanner stage 4 or more has been achieved. This data also suggests that if pubertal stage predicts expected growth hormone response, individualized thresholds based on pubertal stage could be used to negate the need for sex steroid priming, although this has not been directly tested.

In conclusion, there is evidence that sex steroid priming before GHST does impact absolute GH result, the number of children that are considered to have a ‘normal’ GH response, and identifies children who will respond to GH treatment in terms of increase in final height. Future studies to determine optimal priming regimen(s) would be beneficial. Consensus on a widely used and accepted peak GH cut‐off response would also be beneficial. We would recommend subjects in future prospective studies should also be followed up to final height to allow assessment of impact on auxological outcomes.

## AUTHOR CONTRIBUTIONS

Rod T. Mitchell conceived and designed the study. Rod T. Mitchell and Gregor Duncan performed the literature search, data collection and analysis. Sarah Kiff provided critical discussion of the interpreted data. Rod T. Mitchell, Sarah Kiff and Gregor Duncan wrote the manuscript. All authors edited the manuscript and agreed upon the submitted manuscript.

## CONFLICT OF INTEREST

The authors declare no conflict of interest.

## Supporting information

Supporting information.

## Data Availability

The data underlying this article are available in the article and in its online Supporting Information.
