# Supplementary material for: Sex steroid priming for growth hormone stimulation testing in children and adolescents with short stature: A systematic review
Source: Clin Endocrinol (Oxf). 2022 Dec 22;98(4):527–35. doi: 10.1111/cen.14862 (PMC10953312; doi:10.1111/cen.14862)
Supplement: Supplementary file 1 — Supporting information. [file CEN-98-527-s001.docx]

# Supplementary data

**Supplementary Table 1** - Search strategy used for systematic literature search.

| **POPULATION** | Child OR Children OR Adolescent OR Adolescents OR Adolescence OR Teen OR Teens OR Teenager OR Teenagers OR Youth OR Youths OR Puberty OR Pubertal OR Prepuberty OR Prepubertal OR Peripuberty OR Peri-puberty OR Peripubertal |
| --- | --- |
|  | **AND** |
|  | Growth Hormone OR GH OR Growth Hormone Deficiency OR GHD |
| **AND** |  |
| **INTERVENTION** | Priming OR Primer OR Prime OR Primed |
|  | **AND** |
|  | Sex Steroid OR Sex Steroids OR Sex Hormone OR Sex Hormones OR Oestrogen OR Estrogen OR Testosterone |
| **AND** |  |
| **OUTCOME** | Insulin Tolerance Test OR ITT OR Insulin OR Glucagon Stimulation Test OR Glucagon OR Arginine Stimulation Test OR Arginine OR Clonidine Stimulation Test OR Clonidine |

**Supplementary Table 2** – Papers excluded after review of full-text articles with reason for exclusion.

| **Study** | **Reason for Exclusion** |
| --- | --- |
| Binder et al. 2019 | Audit study |
| Chesover et al. 2016 | Survey study |
| Chinoy et al. 2016 | Literature review |
| Coutant et al. 2001 | Not relevant |
| Coutant et al. 2004 | Not relevant |
| Drop et al. 1982 | No full paper available |
| Freeichs et al. 2016 | Survey study |
| Gonc et al. 2011 | Literature summary |
| Hamilton et al. | Conference abstract |
| Hawkes et al. 2016 | Survey study |
| Lazar et al. 2010 | Literature review |
| Lodefalk et al. 2017 | Guideline comment |
| Poyrazoglu et al. 2015 | Survey study |
| Rakhlmova et al. 2017 | Conference abstract |
| Richmond et al. 2008 | Literature review |
| Rosenbloom et al. 2011 | Literature review |
| Ross et al. 1987 | No full paper available |
| Thakur et al. 2017 | Conference abstract |

**Supplementary Table 3** - Risk of bias summary for randomized studies included in this review.

|  | Bias arising from the randomisation | Bias due to deviations from the intended interventions (effect of assignment to intervention) | Risk of bias due to deviations from the intended interventions (effect of adhering to intervention) | Bias due to deviations from intended interventions | Bias due to missing outcome data | Bias in measurement of the outcome | Bias in selection of reported result |
| --- | --- | --- | --- | --- | --- | --- | --- |
| Wilson et al, 1993 |  |  |  |  |  |  |  |
| Marin. 1994 |  |  |  |  |  |  |  |
| Martinez et al. 2000 |  |  |  |  |  |  |  |
| Soliman et al. 2014 |  |  |  |  |  |  |  |

Green – low risk of bias

Amber – moderate risk of bias

Red – high risk of bias

NI – no information available

**Supplementary Table 4** - Risk of bias summary for non- randomized studies included in this review.

|  | Bias due to confounding | Bias in selection of participants into the study | Bias in the classification of interventions | Bias due to deviations from intended interventions | Bias due to missing data | Bias in measurement of outcomes | Bias in selection of reported results |
| --- | --- | --- | --- | --- | --- | --- | --- |
| Moll et al, 1986 |  |  |  |  |  | NI |  |
| Chatterjee et al. 1993 |  |  |  |  |  | NI |  |
| Gonc et al. 2001 |  |  |  |  |  | NI |  |
| Chemaitilly et al. 2003 |  |  |  |  |  | NI |  |
| Muller et al. 2004 |  |  |  |  |  |  |  |
| Couto-Silva et al. 2005 |  |  |  |  |  |  |  |
| Borghi et al. 2006 |  |  |  |  |  |  |  |
| Gonc et al. 2008 |  |  |  |  |  |  |  |
| Molina et al. 2008 |  |  |  |  |  |  |  |
| Sato et al. 2020 |  |  |  |  |  |  |  |
| Galazzi et al. 2020 |  |  |  |  |  |  |  |

Green – low risk of bias

Amber – moderate risk of bias

Red – high risk of bias

NI – no information available

| **Supplementary Table 5** – Detailed summary of studies included in the review. GHST – Growth Hormone Stimulation Test. RCT – Randomised Controlled Trial. ITT – Insulin Tolerance Test. ISS – Idiopathic Short Stature. IM – intramuscular. HV – Height Velocity. BA – Bone Age. TV – Testicular Volume. SDS –Standard Deviation Score. IH – Initial Height. TH – Target Height. | | | | | | |
| --- | --- | --- | --- | --- | --- | --- |
|  |  |  |  |  |  |  |
| **Sex Steroid and Formulation** | **Study** | **Level of Evidence*** | **Population** | **GHST** | **Priming Regimen** | **Findings** |
| **Oestrogen - Oral** | Martinez et al 2000 | 1 | n=59  Age 4-17 yrs  Tanner stage 1/2  15 GHD  44 Short ‘healthy’  - height <- 2SDS | Variable  - Arginine  - Clonidine | Estradiol Valerate  Children <20kg  – 1mg for 3 days  Children >20kg  – 2mg for 3 days | Priming increased mean peak GH response to GHST |
|  | Marin et al 1994 | 1 | n = 84  Tanner stage  - 1 (n = 29)  - 2 (n = 16)  - 3 (n = 18)  - 4 (n = 14)  - 5 (n = 18)  Height  - 2.5-97.5^th^ centile  Tanner stage  - normal for age | Variable  - Arginine  - ITT  - Exercise | Ethinyl Estradiol  40µg/m^2^  - divided into 3 doses for 2 days | Priming increased mean peak GH response to GHST |
|  | Wilson et al 1993 | 1 | n=65  Age – 1-15 yrs  Height  - <5^th^ centile (+/- delayed BA)  Tanner stage  – not reported | Variable  - Arginine  - ITT  - Clonidine | Conjugated Estrogen  (Premarin)  2.5mg  - 2 doses  (evening before & morning of test) | Priming did not increase GH response to GHST |
|  | Moll et al 1986 | 1 | n=23  Age 3-15 yrs  Height  - <3^rd^ centile  Tanner stage  - prepubertal | Levodopa | Ethinyl Estradiol  7-40µg/m^2^/d  - Single pill day 1  - Split dose day 2 (prior to testing) | Priming increased the number of test subjects reaching cut-off GH response of about 7 μg/L |
| **Oestrogen Patch** | Borghi et al 2006 | 2 | n=22  Age 3-13 yrs  ISS  Height  - < target height with bone age delay  Tanner stage  – not reported | Clonidine | Ethinyl Estradiol  50µg/d  - 3 days | Priming increased GH response to GHST |
| **Testosterone IM** | Gonc et al 2008 | 3 | n=50 (boys)  Age 10-16 yrs  Height  - Short stature  - HV (<5cm/yr)  - BA delay  Tanner stage  - 1 (n=23)  - 2 (n=27) | Levodopa | Testosterone Esters  (Sustanon)  Low dose  - 62.5mg/m^2^  High dose  - 125mg/m^2^  Multiple dose  - 62.5mg/m^2^ x3  ^-^ 4 weeks apart  Priming completed 7d prior to test | 50 boys failed unprimed but responded to primed GHST.  Final adult height was similar to mid- parental height in these boys |
|  | Couto-Silva et al 2005 | 3 | n=148 (boys)  Age >14 yrs  Height  - Short stature  - <-2 SDS  Tanner stage  - delayed puberty | Arginine  ITT | Testosterone Heptylate  100mg x2-4  - 15 days apart | Priming increased GH response to GHST |
|  | Chemaitilly et al 2003 | 3 | n=47 (boys)  Age 0-17 yrs  Height  - Short stature  - <-2 SDS  Tanner stage 1/2 | Variable  - Arginine  - ITT  - Glucagon  - Ornithine | Testosterone Heptylate  100mg (2 doses)  - 15 days apart  - complete 10d prior to testing | Priming increased mean peak GH response during sleep testing but not upon GHST |
|  | Gonc et al 2001 | 2 | n=84 (boys)  Age 10-17 yrs  Height  - Short stature  - HV (<25^th^ centile)  Tanner stage1/2 | Levodopa | Testosterone Esters  (Sustanon)  Low dose  - 62.5mg/m^2^  High dose  - 125mg/m^2^  Multiple dose  - 62.5mg/m^2^ (3 doses)  ^-^ 4 weeks apart  Priming completed 7d prior to test | Low and High dose  - equally effective in increasing GH response.  Multiple dose  - useful in those who have failed GHST on single dose priming |
|  | Muller et al 2004 | 2 | n=26 (boys)  Age 14.3 +/- 1.1 yrs  Height  - Short stature  - HV (<25^th^ centile)  Tanner stage1/2 | Arginine | Testosterone Enanthate  100mg  - 3-10 days prior to test | Priming increased mean peak GH response to GHST |
|  | Chaterjee et al 1993 | 2 | n=28 (boys)  B-Thalassaemia with pubertal delay  Age >14 yrs  Height not reported  Tanner stage1 | ITT | Testosterone Esters  (Sustanon)  100mg  - 5 days prior to test | Priming did not increase GH response to GHST |
|  | Sato et al 2020 | 3 | n=3 (boys)  Age 13-14 yrs  Height  - Short stature  Tanner stage  - prepubertal (TV<4mls) | Variable  - Arginine  - ITT | Testosterone Enanthate  100mg  - 7 days prior to test | Priming increased GH response to GHST |
| **Mixed Regimens**  - Testosterone IM (boys)  - Oestrogen - Oral  (girls) | Galazzi et al 2020 | 3 | n=184  Age  - 11-14 yrs (boys)  - 10-13 yrs (girls)  Height  - Short stature  - IH-TH < -1.5 SDS  - HV < -1 SDS  Tanner stage1/2 | Variable  - Arginine  - ITT  - Glucagon  - Clonidine | Boys  Testosterone Enanthate or Proprionate (4-7d)  - Low dose – 50mg  - High dose – 100mg  Girls  Ethinyl Estradiol (Oral)  - 100µg/d (3 doses)  or  Stilbestrol  - 1mg twice daily (2 days) | Priming played a key role in identifying children who may benefit most from recombinant GH treatment |
|  | Soliman et al 2014 | 1 | n=92  Age 9-13 yrs  Height  - Short stature  - < -2SDS  Tanner stage  - prepubertal | Clonidine | Boys  Testosterone depot  - 25mg  - 7-10 days prior to test  Girls  Conjugated Estrogens  - 1.25mg/d (3 days) | Priming did not increase GH response to GHST |
|  | Molina et al 2008 | 2 | n=39  Age 12 +/- 2 yrs  Height  - Short stature  - < -2SDS  or  - HV< -1SDS  Tanner stage1/2 | Clonidine | Boys  Testosterone Enanthate  - 100mg  - 5-8 days prior to test  Girls  Estradiol Valerate  - 1mg/d (3 days) |  |

*Level of Evidence (adapted from https://[www.cebm.net/2016/05/ocebm-levels-of-evidence/).](http://www.cebm.net/2016/05/ocebm-levels-of-evidence/))

1—Properly powered and conducted randomised clinical trial; systematic review with meta-analysis.

2—Well-designed controlled study without randomisation; prospective comparative cohort study.

3—Case–control studies; retrospective cohort study.

4—Case series with or without intervention; cross-sectional study.

5—Opinion of respected authorities; case reports

**Supplementary Table 6** – Detailed summary of studies comparing multiple dosing regimens. GHST – Growth Hormone Stimulation Test. ITT – Insulin Tolerance Test. IM – intramuscular. HV – Height Velocity. SDS –Standard Deviation Score Height. IM – intra-muscular.

| **Study** | **Level of Evidence*** | **Population** | **GHST** | **Sex Steroid and Formulation** | **Priming Regimen** | **Findings** |
| --- | --- | --- | --- | --- | --- | --- |
| Moll et al 1986 | 1 | n=23  Age 3-15 yrs  Height  - <3^rd^ centile  Tanner stage  - prepubertal | Levodopa | Ethinyl Estradiol - Oral | 7-40µg/m^2^/d  - Single pill day 1  - Split dose day 2 (prior to testing)  No single or double dose | Peak GH response to levodopa GHST were significantly higher following two oestrogen doses compared to one. |
| Couto-Silva et al 2005 | 3 | n=148 (boys)  Age >14 yrs  Height  - Short stature  - <-2SDS  Tanner stage  - delayed puberty | Arginine  ITT | Testosterone Heptylate - IM | No dose  or  100mg (x2)  or  100mg (x4)  - 15 days prior to testing | Priming increased GH response to GHST |
| Gonc et al 2001 | 2 | n=84 (boys)  Age 10-17 yrs  Height  - Short stature  - HV (<25^th^ centile)  Tanner stage1/2 | Levodopa | Testosterone Esters - IM  (Sustanon) | Low dose  - 62.5mg/m^2^  High dose  - 125mg/m^2^  Multiple dose  - 62.5mg/m^2^ (3 doses)  ^-^ 4 weeks apart  Priming completed 7d prior to test | Low and High dose  - equally effective in increasing GH response.  Multiple dose  - useful in those who have failed GHST on single dose priming |

*Level of Evidence (adapted from https://[www.cebm.net/2016/05/ocebm-levels-of-evidence/).](http://www.cebm.net/2016/05/ocebm-levels-of-evidence/))

1—Properly powered and conducted randomised clinical trial; systematic review with meta-analysis.

2—Well-designed controlled study without randomisation; prospective comparative cohort study.

3—Case–control studies; retrospective cohort study.

4—Case series with or without intervention; cross-sectional study.

5—Opinion of respected authorities; case reports.
